# Effect of the selection pressure of vaccine antibodies on evolution of H9N2 avian influenza virus in chickens

**DOI:** 10.1186/s13568-020-01036-0

**Published:** 2020-05-27

**Authors:** Hailong Su, Yu Zhao, Lirong Zheng, Shifeng Wang, Huoying Shi, Xiufan Liu

**Affiliations:** 1 College of Veterinary Medicine, Yangzhou University, Yangzhou, 225009 Jiangsu China; 2 Department of Infectious Diseases and Immunology, College of Veterinary Medicine, University of Florida, Gainesville, FL 32611-0880 USA; 3 College of Veterinary Medicine, Yangzhou University, Yangzhou, 225009 Jiangsu People’s Republic of China; 4 Key Laboratory of Avian Preventive Medicine, Ministry of Education, Yangzhou University, Yangzhou, China; 5 Jiangsu Key Laboratory of Zoonosis, Yangzhou University, Yangzhou, 225009 Jiangsu China; 6 Jiangsu Co-innovation Center for Prevention and Control of Important Animal Infectious Diseases and Zoonosis, Yangzhou, 225009 Jiangsu China

**Keywords:** H9N2 avian influenza virus, Evolution, Selection pressure of vaccine antibodies, Antigenic variation

## Abstract

H9N2 avian influenza virus has spread worldwide, and vaccination with an inactivated virus is currently the major prevention method in China. To further understand the effect of the selection pressure from antibodies on the evolution of H9N2 avian influenza virus, F/98 (A/Chicken/Shanghai/F/98), which is the vaccine representative of H9N2 avian influenza virus in East China, was used for serial passaging for 20 generations in chickens with and without vaccination. After plaque purification from trachea and lung tissues, 390 quasispecies were obtained. The second-generation quasispecies under the selection pressure of vaccine antibodies had undergone 100% antigen variation, while after passaging to the fifth generation, only 30–40% of the quasispecies displayed antigen variation when there was no selection pressure of vaccine antibodies, implying that the selection pressure of vaccine antibodies promotes the antigen variation of F/98. We found for the first time that there were three mutation hotspots in the HA genes of the quasispecies under the selection pressure of vaccine antibodies, which were K131R, A168T, and N201D. Moreover, under the selection pressure of vaccine antibodies, 10 amino acids (67–76) of the NA protein of all quasispecies were deleted, and PB2 of the quasispecies had undergone a high-frequency R355K mutation. However, without selection pressure of vaccine antibodies, NP had undergone two high-frequency mutations, namely, V186I and L466I, and a high-frequency mutation of L77I appeared in the NS gene. This result shows that the vaccine antibody selection pressure could control and regulate gene variation of the F/98 virus. Compared to that of the parental virus F/98, the EID_50_ of the twentieth passaged virus under the selection pressure of vaccine antibodies did not change, while the EID_50_ of the twentieth passaged virus without selection pressure of vaccine antibodies was significantly enhanced by 794 times. Furthermore, the twentieth passaged virus with selection pressure from vaccine antibodies lost its lethal ability in embryonated chicken eggs, whereas the EID_50_ of the twentieth passaged virus without selection pressure of vaccine antibodies increased to 6.3 times that of the F/98 strain. All the above results show that the selection pressure of vaccine antibodies promotes the antigen variation of H9N2 avian influenza virus and plays a role in regulating and controlling gene mutation of H9N2 avian influenza virus.

## Introduction

In 1998, infectious cases of H9N2 avian influenza were found in East China and were determined to be due to the A/Chicken/Shanghai/F/98 (F/98) strain. Afterward, F/98 has spread in East China, and it has gradually evolved into many branches (Chang et al. 2018; Chen et al. 2006, 2013; Gu et al. 2017). To control the spread and infection of H9N2 subtype avian influenza, the government used inactivated F/98 as a vaccination to control H9N2 subtype avian influenza virus (Gu et al. 2017; Zhang et al. 2008). However, the virus still spread in most provinces of China (Zhang et al. 2008). According to some reports, there were still some infectious cases occurring in animals that were preimmunized and had high levels of antibodies (Lee and Suarez 2005). In a practical test, it was found that there were many cases of viral recombination and that the H9N2 virus had undergone obvious antigen variation in a chicken farm with vaccinations. Compared with that of the strains in previous years, the new virus’s ability to duplicate was greatly enhanced (Zhang et al. 2008).

The influenza virus mutates its genes to obtain the ability to adapt to its host, while the high mutation rate for the virus provides a feasible condition for the process (Nelson et al. 2014; Petrova and Russell 2018; Tewawong et al. 2017). Regarding the H5N2 subtype avian influenza virus, HA generated a Q234L mutation after many generations in the lungs of mice, which helped to the virus obtain the ability to infect mammals (Petrova and Russell 2018). 627 K and 701 N mutations in the PB2 protein affected the toxicity and host range of influenza viruses (Sang et al. 2015; Sediri et al. 2015). After serial passaging in guinea pigs, four high-frequency hotspots (75–85, 125–135, 165–170, and 225–230) were identified in the HA of H3N2 subtype influenza virus (Long et al. 2011). With these mutations, H3N2 subtype influenza virus generated antigen drift and escaped from the neutralization of host-specific antibodies. After serial passaging in immunized ferrets, the internal genes of H1N1 generated corresponding mutations to adapt to the immune state in the host (Guarnaccia et al. 2013). After serial passaging in pigs, H9N2 subtype avian influenza virus had an increased duplication ability (Mancera Gracia et al. 2017). Mutation of H5N1 virus isolated from a chicken after H5N1 vaccination occurred in mainly the adjacent areas of antigen loci and the relevant site (Abdel-Moneim et al. 2011). At present, there have been no reports on the effect of the selection pressure of vaccine antibodies in host chickens on the evolution of H9N2 avian influenza virus. However, the host chicken is both the natural host of avian influenza virus and possibly the terrestrial poultry able to make the terrestrial poultry avian influenza virus infectious to humans. Therefore, it was extremely necessary to research the effect of the selection pressure of vaccine antibodies in host chickens on the mutation of H9N2 avian influenza virus.

In this study, 4-week-old specific pathogen-free (SPF) chickens were used as infectious models, and SPF chickens immunized with the F/98 inactivated vaccine were infected with the F/98 strain virus. Then, the model for serial passaging of H9N2 subtype avian influenza with selection pressure of vaccine antibodies was established. The antigenic variation of offspring viruses with and without selection pressure of vaccine antibodies was compared after the viruses were purified by plaque purification. Moreover, the gene sequence of the offspring viruses obtained after isolation was also analyzed to explore the effect of selection pressure of vaccine antibodies on the evolution of H9N2 avian influenza virus.

## Materials and methods

### Virus and cells

F/98 was identified as H9N2 subtype avian influenza virus by the Key Open Lab of Animal Infectious Diseases of the Agricultural Ministry (Yangzhou, China). The virus was passaged using SPF chicken embryos and then stored at − 80 °C. Madin–Darby canine kidney (MDCK; ATCC, CCL34) cell line cells were grown in Dulbecco’s modified Eagle medium (DMEM; Gibco, USA) supplemented with 10% fetal bovine serum (HyClone, Australia), 100 IU/mL penicillin (Sigma, USA), and 100 μg/mL streptomycin (Sigma, USA).

### Vaccine preparation

The F/98 strain was inactivated by adding 0.2% formalin (v/v) for 24 h at 37 °C. Inactivation was confirmed by the absence of detectable infectivity after two blind passages of formalin-treated allantoic fluid in embryonated eggs. The inactive allantoic fluid was emulsified in two parts of paraffin oil (v/v), which is currently used commercially as an adjuvant for veterinary vaccine production.

### Hemagglutination inhibition (HI) assays

To begin, 25 μL of phosphate-buffered saline (PBS) was added to every hole of a reaction plate. Then, 25 μL of serum was added into the first hole of the first column and mixed several times. Two-fold dilutions of 0.025 mL volumes of the serum were used across the plate. Next, 4 hemagglutinating units (HAU) of virus in 25 μL was added to each well and left for a minimum of 30 min at room temperature. Finally, 25 μL of 1% (v/v) chicken red blood cells (RBCs) were added to each well and mixed gently, allowing the RBCs to settle for approximately 40 min at room temperature, by which time control RBCs should have formed a distinct button. The HI titer was the highest dilution of serum causing complete inhibition of 4 HAU of antigen.

### Antisera preparation of F/98 virus

Six-week-old SPF chickens were immunized by intramuscular injection of 1 mL of an oil emulsion of inactivated whole-virus vaccines of representative viruses for 4 weeks. Then, chickens were bled. Sera were treated with receptor-destroying enzyme (RDE, Sigma, USA) to remove nonspecific hemagglutination inhibitors, as described previously, before an HI test was performed (Ninomiya et al. 2002).

### Serial passaging of F/98 strain virus with and without selection pressure of vaccine antibodies

**Fig. 1:**
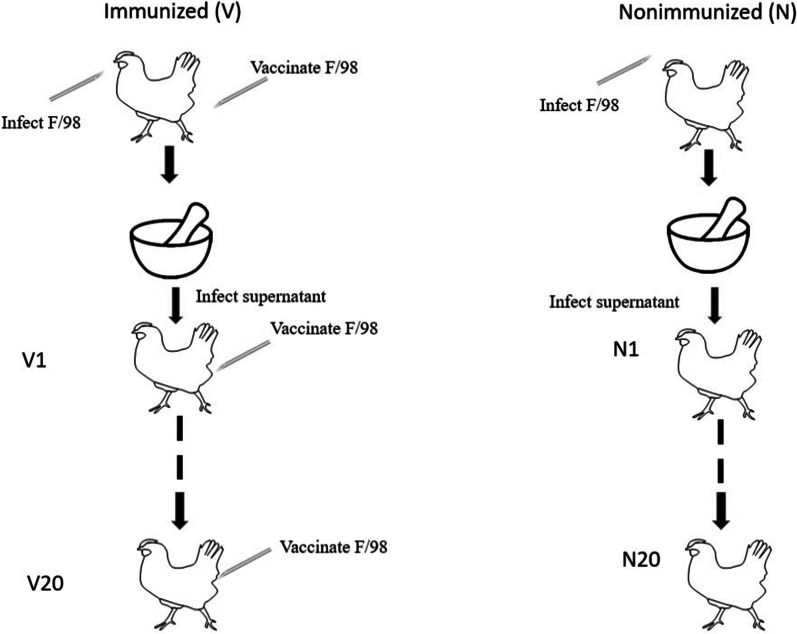
The schematic diagram for F/98 serial passaging under selective pressure with and without vaccine antibodies

Four-week-old SPF chickens were immunized with emulsified F/98 strain virus vaccine (1 mL/chicken), and after completion, the blood of chickens was collected every 12 h. When the serum HI titer of the immunized chickens reached 40 or above, it showed that the neutralizing antibody for the F/98 strain virus had already been generated within the chickens, with effective selection pressure of vaccine antibodies being generated as well. Three vaccinated SPF chickens were injected with 10^6^ 50% egg infectious dose (EID_50_) of F/98 virus by nasal drop, eye drop, laryngeal injection and so on. After 3 days of infection, the chickens were sacrificed by euthanasia, and then the trachea and lung tissues of the chickens were taken. The trachea and lung tissues collected were weighed, and four units of antigen PBS was added at a ratio of 1:10 with the ground trachea and lung tissues. The ground tissue liquid was processed at 4 000 rpm in a 4 °C environment, with the liquid supernatant obtained containing the first generation of virus. The liquid supernatants of the trachea and lung tissues were mixed at a ratio of 1:1. Then, 200 µL was taken to infect the second batch of SPF chickens according to the abovementioned method, and the liquid supernatant with the second generation of virus was also obtained. The process of serial passaging of the F/98 strain with selection pressure of vaccine antibodies was repeated until the liquid supernatant of the twentieth generation virus was obtained, which is shown in Fig. 1. The continuous generation passaging was carried out until the twentieth generation was completed with the ground lung tissue liquid marked as 1VL–20VL and the ground trachea tissue liquid marked as 1VB–20VB (Fig. 1).

Regarding the serial passaging of generations without selection pressure of vaccine antibodies, the serum of SPF chickens was taken, and the F/98 virus was used for the HI test under the condition of nonimmunization. When the HI titer was 0, three SPF chickens were injected with 10^6^ EID_50_ of F/98 virus by nasal drop, eye drop, laryngeal injection and so on. After 3 days of infection, the chicken was sacrificed by euthanasia, and then trachea and lung tissues of the chickens were taken. The trachea and lung tissues collected were weighed, and four units of antigen PBS was added at a ratio of 1:10 with the ground trachea and lung tissues. The ground tissue liquid was processed at 4000 rpm in a 4 °C environment, with the liquid supernatant obtained containing the first generation of virus. The liquid supernatants of the trachea and lung tissues were mixed at a ratio of 1:1. Then, 200 µL was taken to infect the second batch of SPF chickens according to the abovementioned method, and the liquid supernatant with the second generation of virus was also obtained. The process of serial passaging of the F/98 strain with selection pressure of vaccine antibodies was repeated until the liquid supernatant of the twentieth generation virus was obtained, which is shown in Fig. 1. The continuous generation passaging was carried out until the twentieth generation was completed, with the ground lung tissue liquid marked as 1NL–20NL and the trachea tissue ground liquid marked as 1NB–20NB (Fig. 1).

### Plaque assays

Plaque assays were used for purification of virus isolates propagated in the chicken. Briefly, 6-well plates were seeded with 3 × 10^4^ MDCK cells. The following day, serial dilutions of influenza virus were applied in 1 mL volumes for 1 h. The monolayers were washed once with PBS and overlaid with a solution of 1% agarose in DMEM supplemented with 0.1% neutral red. Clear plaques were visible against the stained cells after 3–4 days of incubation at 37 °C.

### Assay of viral antigenic drift

Plaque-purified virus was prepared as 4 units of HA and used to carry out the HI test with the F/98 serum processed by RDE, and the HI results obtained were compared with those for the F/98 maternal virus. When the difference was larger than 4, it was categorized as antigen variation.

### Eid_50_/eld_50_

To test the changes in toxicity, we consecutively diluted the virus to be tested 10 times and then took 200 µL to inject into a 10-day-old SPF chick embryo; each virus was injected into 5 chick embryos. The embryo was observed every 12 h, and the time for the death of an embryo was recorded. After 120 h, the allantoic fluid was collected to test the HA titer and calculate EID_50_ and 50% embryo lethal dose (ELD_50_) according to the method of (Reed and Muench 1938).

### Sanger sequencing

Genome RNAs of viral clones were extracted from culture supernatants using a QIAamp viral RNA kit (Qiagen). Genes were reverse transcribed and amplified using a OneStep RT-PCR kit (Qiagen). The primers used in this study are listed in Table 1. The amplified cDNA products were excised from agarose gels and purified using a QIAquick gel extraction kit (Qiagen). Full-genome DNAs were Sanger sequenced by TAKARA BIOTECHNOLOGY (DALIAN, China) CO., LTD., and the sequence data were analyzed using GenScan software.

**Table 1:** Primer sets for amplification of the whole genome of F/98

Prime name	Prime sequence (5′–3′) of forward primer	Prime sequence (5′-3′) of reverse primer	Length (bp)
PB2			
PB2-1	TATTGGTCTCAGGGAGCGAAAGCAGGTC	ACTGCCTTTATCATGCAGTCATC	1376
PB2-2	GCAACCGCTATTTTTAGGAAAGC	ATATGGTCTCGTATTAGTAGAAACAAGGTCG	1276
PB1			
PB1-1	AGCAAAAGCAGGCAAACCATTTGAA	GAGGATTGGAGCCCTTCCCACCA	1984
PB1-2	ATGATGATGGGCATGTTCAACATG	AGTAGAAACAAGGCATTTTTTCA	1120
PA			
PA-1	AGCTAAAGCAGGTACTGATTCAAAA	ATCCATCTTGATTCCGTCAATTC	1316
PA-2	GGCACCTGATAAAGTTGACTTTGA	AGTAGAAACAAGGTACTTTTTTGGA	1138
HA	TATTCGTCTCAGGGAGCAAAAGCAGGGG	ATATCGTCTCGTATTAGTAGAAACAAGGGTGTTTT	1683
NP	TATTCGTTCTCAGGGAGCAAAAGCAGGGTA	AGTAGAAACAAGGGTATTTTTCTT	1497
NA	TATTGGTCTCAGGGAGCAAAAGCAGGAGT	ATATGGTCTCGTATTAGTAGAAACAAGGAGTTTTT	1417
M	TATTCGTCTCAGGGAGCAAAAGCAGGTAG	ATATCGTCTCGTATTAGTAGAAACAAGGTAGTTTT	982
NS	TATTCGTCTCAGGGAGCAAAAGCAGGGTG	ATATCGTCTCGTATTAGTATAAACAAGGGTGTTTT	844

### Protein 3D structure

The positions of amino acid substitutions on the HA molecule were analyzed on the 3-dimensional structure obtained from the Protein Databank (PDB, accession number 1JSD) with the RasMol 2.7.3 program.

### Multiple sequence analysis

Comparative analyses were performed using the CLUSTAL W multiple sequence alignment program, Mega7.1. Representative avian influenza virus sequences used for the alignments were obtained from the GenBank and EMBL databases.

### Statistics

Data are presented as the geometric means and standard deviations for all assays. The Mann–Whitney U test (GraphPad Software, Inc., San Diego, CA) was used to evaluate the replication ability of strains and the tissue lesions in chickens challenged with different viruses. A P value of 0.05 was considered statistically significant.

## Results

### F/98 serial passaging with and without selection pressure of vaccine antibodies

At present, the major prevention and control measure for H9N2 subtype avian influenza virus relies on mainly the whole-virus inactivated vaccine (Gu et al. 2017). The inactivated vaccine is able to induce humoral immunity in chickens (Chen and Deng 2009). However, H9N2 subtype avian influenza virus has spread among the chickens of China, and the H9N2 subtype avian influenza virus can still be isolated from chicken flocks with high vaccine antibodies. One of the reasons is possibly the effect of the selection pressure of vaccine antibodies. To explore the effect of homologous selection pressure of vaccine antibodies on the evolution of H9N2 subtype avian influenza virus, this study adopted 4-week-old SPF chickens for use in an animal model with selection pressure of vaccine antibodies, and these naive chickens were called nonimmunized chickens; an F/98 strain oil emulsion vaccine was used to immunize 4-week-old SPF chickens, and the ones whose serum contained a 40–320 HI titer of F/98 strain virus were regarded as animals with selection pressure of vaccine antibodies, namely, the immunized chickens (Fig. 1). In the 1–10 passaging generations, the HI titer in the serum of immunized chickens was controlled within 40 (Table 2). In the 11–20 passaging generations, the HI titer in the sera of immunized chickens was controlled within 320 (Table 2). In the first passage generation, 10^6^ EID_50_ of the F/98 strain virus was used to infect three immunized and nonimmunized chickens by nasal drop, eye drop and intratracheal injection. On the 3rd day after infection, the chickens were sacrificed, and trachea and lung tissues were collected and added into PBS according to a mass/volume ratio of tissue to PBS of 1:10. Under aseptic conditions, the tissues were ground completely, with the supernatant being collected. Afterward, the liquid was divided into three parts. One part was used to vaccinate a 10-day-old SPF chick embryo to test the passaged virus from the trachea and lung tissues. The other part was reserved in a − 80 °C freezer for purification of quasispecies viruses; then, 100 μL of supernatant from the trachea and lung tissues was collected and mixed as the seed virus to infect the next generation of immunized or nonimmunized chickens. After passaging the virus for 20 generations in the immunized or nonimmunized chickens, purification of quasispecies viruses was carried out for the supernatant from trachea and lung tissues of immunized and nonimmunized chickens from the 1–5, tenth, fifteenth and twentieth generations by plaque isolation. Viruses obtained from plaques were propagated in 10-day-old SPF chick embryos, and the allantoic fluid was tested with 1% red blood cells. The HA-positive allantoic fluid was stored in a − 80 °C freezer. Consequently, 390 progeny strains of virus were isolated (Table 3). One percent red blood cells were used to test the liquid, and the results show that all of the HA titers of purified quasispecies viruses were within 6–10 log2.

**Table 2:** Antibody titers in chicken sera in this study

Generation	Immunized			Nonimmunized		
	Chicken-1	Chicken-2	Chicken-3	Chicken-1	Chicken-2	Chicken-3
1	40	40	40	0	0	0
2	40	40	40	0	0	0
3	40	40	40	0	0	0
4	40	40	40	0	0	0
5	40	40	40	0	0	0
6	40	40	40	0	0	0
7	40	40	40	0	0	0
8	40	40	40	0	0	0
9	40	40	40	0	0	0
10	40	40	40	0	0	0
11	320	320	320	0	0	0
12	320	320	320	0	0	0
13	320	320	320	0	0	0
14	320	320	320	0	0	0
15	320	320	320	0	0	0
16	320	320	320	0	0	0
17	320	320	320	0	0	0
18	320	320	320	0	0	0
19	320	320	320	0	0	0
20	320	320	320	0	0	0

**Table 3:** The count of quasispecies viruses isolated from lung and trachea tissues with and without vaccine selective pressure

Generation	Strain count	Generation	Strain count	Generation	Strain count
1VB	10	4VB	10	15VB	10
1VL	12	4VL	11	15VL	10
1NB	11	4NB	12	15NB	13
1NL	10	4NL	10	15NL	11
2VB	10	5VB	17	20VB	20
2VL	10	5VL	11	20VL	24
2NB	10	5NB	16	20NB	19
2NL	10	5NL	10	20NL	15
3VB	10	10VB	10		
3VL	10	10VL	11		
3NB	10	10NB	11		
3NL	10	10NL	10		

### Antigen variation of H9N2 subtype avian influenza virus accelerated by selection pressure of vaccine antibodies

**Fig. 2:**
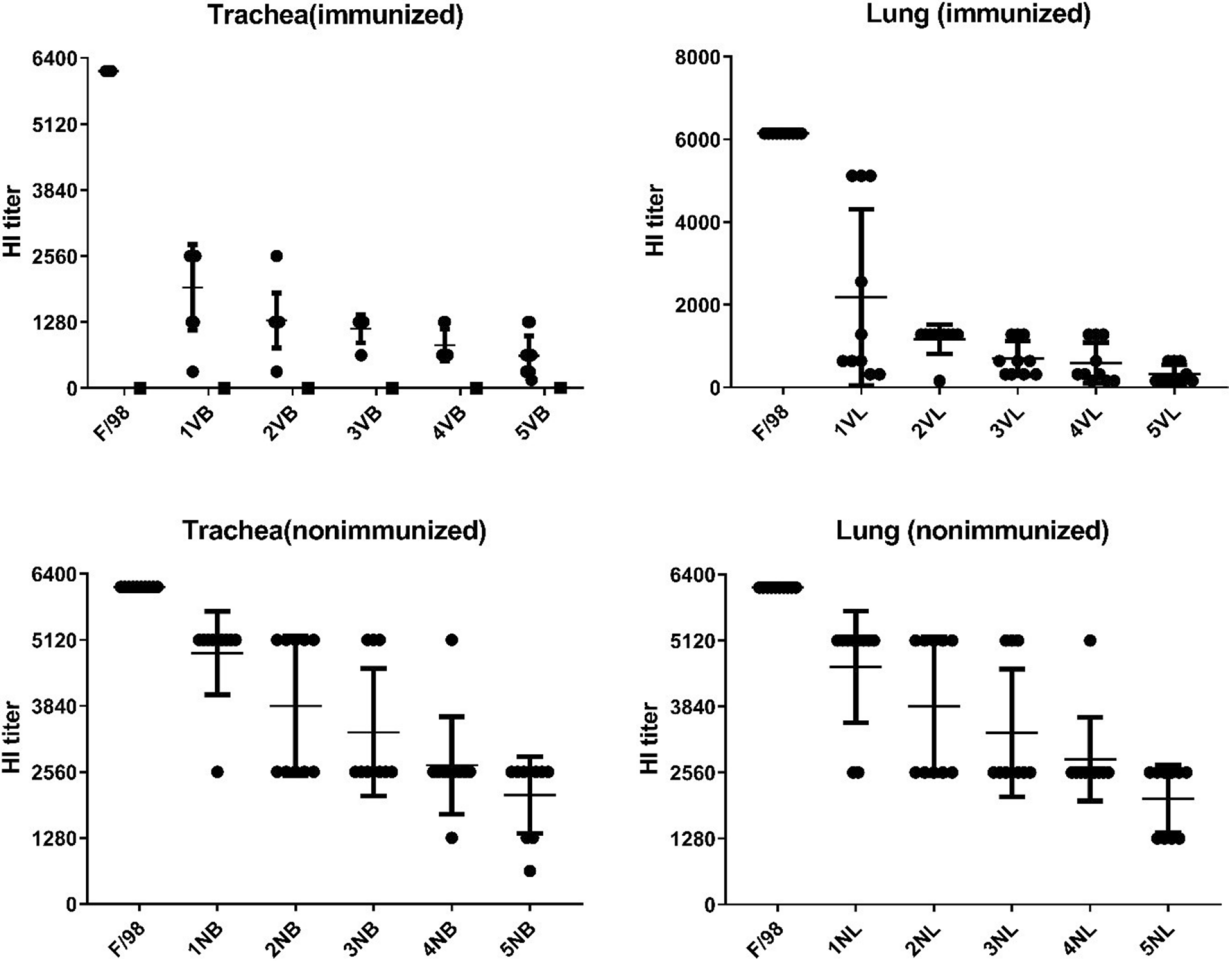
F/98 virus antigen variation under antibody pressure

To explore the effect of the selection pressure of vaccine antibodies on F/98 strain viral antigenicity, the study first used F/98 to test the HI titer of generations 1–5 of purified viruses from the trachea and lung tissues of the immunized and nonimmunized chickens. The results show that the HI titer of F/98 was 6144. The average HI titers of quasispecies viruses of generations 1–5 isolated from trachea tissues with selection pressure of vaccine antibodies were 1952, 1312, 1152, 832 and 624, respectively. The proportion of progeny viruses whose HI titer decreased by more than 4 times in generations 1–5 was 40% (the first generation), 80% (the second generation), 100% (the third generation), 100% (the fourth generation) and 100% (the fifth generation); the average HI titers of quasispecies viruses of generations 1–5 without selection pressure of vaccine antibodies isolated from trachea were 4864, 3840, 3328, 2688 and 2112, respectively. The proportion of progeny viruses whose HI titer decreased by more than 4 times in generations 1–5 was 0% (the first generation), 0% (the second generation), 0% (the third generation), 10% (the fourth generation) and 30% (the fifth generation). The results showed that, in the trachea, when the F/98 strain with selection pressure of vaccine antibodies was passaged for the first generation, there were antigen variant strains, and when it was passaged for the third generation, variation took place in the antigenicity for all the quasispecies viruses. Without selection pressure of vaccine antibodies, only 30% of the quasispecies viruses after the F/98 strain was passaged to the fifth generation had undergone variation (Fig. 2).

The average HI titers of quasispecies viruses of generations 1–5 isolated from lungs with selection pressure of vaccine antibodies were 2176, 1168, 704, 592 and 320, respectively, which were 2.8, 5.3, 8.7, 10.4 and 19.2 times lower than the HI titer of the F/98 strain, respectively. The proportion of progeny viruses whose HI titer decreased by more than 4 times in generations 1–5 was 60% (the first generation), 100% (the second generation), 100% (the third generation), 100% (the fourth generation) and 100% (the fifth generation). The average HI titers of quasispecies viruses of generations 1–5 isolated from lungs without selection pressure of vaccine antibodies were 4608, 3840, 3328, 2816 and 2048, respectively, which were 1.3, 1.6, 8.7, 1.8 and 3 times lower than the HI titer of the F/98 strain, respectively. The proportion of progeny viruses whose HI titer decreased by more than 4 times in generations 1–5 was 0% (the first generation), 0% (the second generation), 0% (the third generation), 0% (the fourth generation) and 40% (the fifth generation). The results showed that, in the lung, when the F/98 strain with selection pressure of vaccine antibodies was passaged for the first generation, there were antigen variant strains, and when it was passaged for the second generation, variation took place in the antigenicity for all the quasispecies viruses. Without selection pressure of vaccine antibodies, only 40% of the quasispecies viruses after the F/98 strain was passaged to the fifth generation had undergone variation (Fig. 2). All the above results showed that the selection pressure of vaccine antibodies was effective in obviously promoting the antigen variation of H9N2 subtype avian influenza virus.

### Selection pressure of vaccine antibodies regulated HA gene mutation of H9N2 subtype avian influenza virus

HA is the major surface protein of avian influenza virus as well as the major antigen protein of the virus. On the HA protein, antigen determinants and receptor-binding sites of the virus are distributed. After infection with avian influenza virus or vaccination with avian influenza virus, specific antibodies for HA protein are generated in the body. In the process of the interaction between influenza and the host, the virus will escape from antibodies by amino acid variation on HA. After passaging the F/98 strain in the chickens for generations with and without selection pressure of vaccine antibodies, the study showed that selection pressure of vaccine antibodies can obviously promote the variation of F/98. To further study the evolution of the HA antigen protein of the F/98 strain when it escapes from the selection pressure of vaccine antibodies, the isolated viruses were sequenced. Comparing to HA gene sequence of F/98, there were 32 nucleotide mutations in the HA sequence of the virus isolated from the trachea with selection pressure of vaccine antibodies, including 19 meaningful mutations, accounting for 43.75% of the total amount; it was found out that there were 32 nucleotide mutations in HA of the virus isolated from the lung with selection pressure of vaccine antibodies, including 14 meaningful mutations, accounting for 57.58% of the total amount; it was found out that there were 32 nucleotide mutations in HA of the virus isolated from the trachea without selection pressure of vaccine antibodies, including 32 meaningful mutations, accounting for 53.13% of the total amount; it was found out that there were 26 nucleotide mutations in HA of the virus isolated from the lung without selection pressure of vaccine antibodies, including 20 meaningful mutations, accounting for 76.92% of the total amount; and it was found out that the meaningful mutation rate of the virus isolated from the lung was higher than that of the virus isolated from the trachea ($, & and P < 0.05, seen in Table 4). These results show that the meaningful mutation rate with selection pressure of vaccine antibodies was positively lower than that without selection pressure of vaccine antibodies (seen in Table 4, P < 0.05) All of the results implied that selection pressure of vaccine antibodies had the effect of limiting the mutation of the HA genes of the F/98 strain in H9N2 subtype avian influenza virus; moreover, the lung had larger effect on the HA genes of the F/98 strain of H9N2 subtype avian influenza virus than the trachea.

**Table 4:** Synonymous and nonsynonymous mutations in the HA protein

Immune status	Tissue	Sequenced HA genes	HA variant sites	Synonymous mutations	Nonsynonymous mutations
Immunized	Trachea	97	32	14 (43.75%)*^$^	18 (56.25%)
Immunized	Lung	99	33	19 (57.58)^#$^	14 (42.42%)
Nonimmunized	Trachea	102	32	17 (53.13%)*^&^	15 (46.87%)
Nonimmunized	Lung	86	26	20 (76.92%)^#&^	6 (23.08%)

*^,$,#,&^P < 0.05

### A special mutation hotspot generated by the H9N2 subtype avian influenza virus with selection pressure of vaccine antibodies

**Fig. 3:**
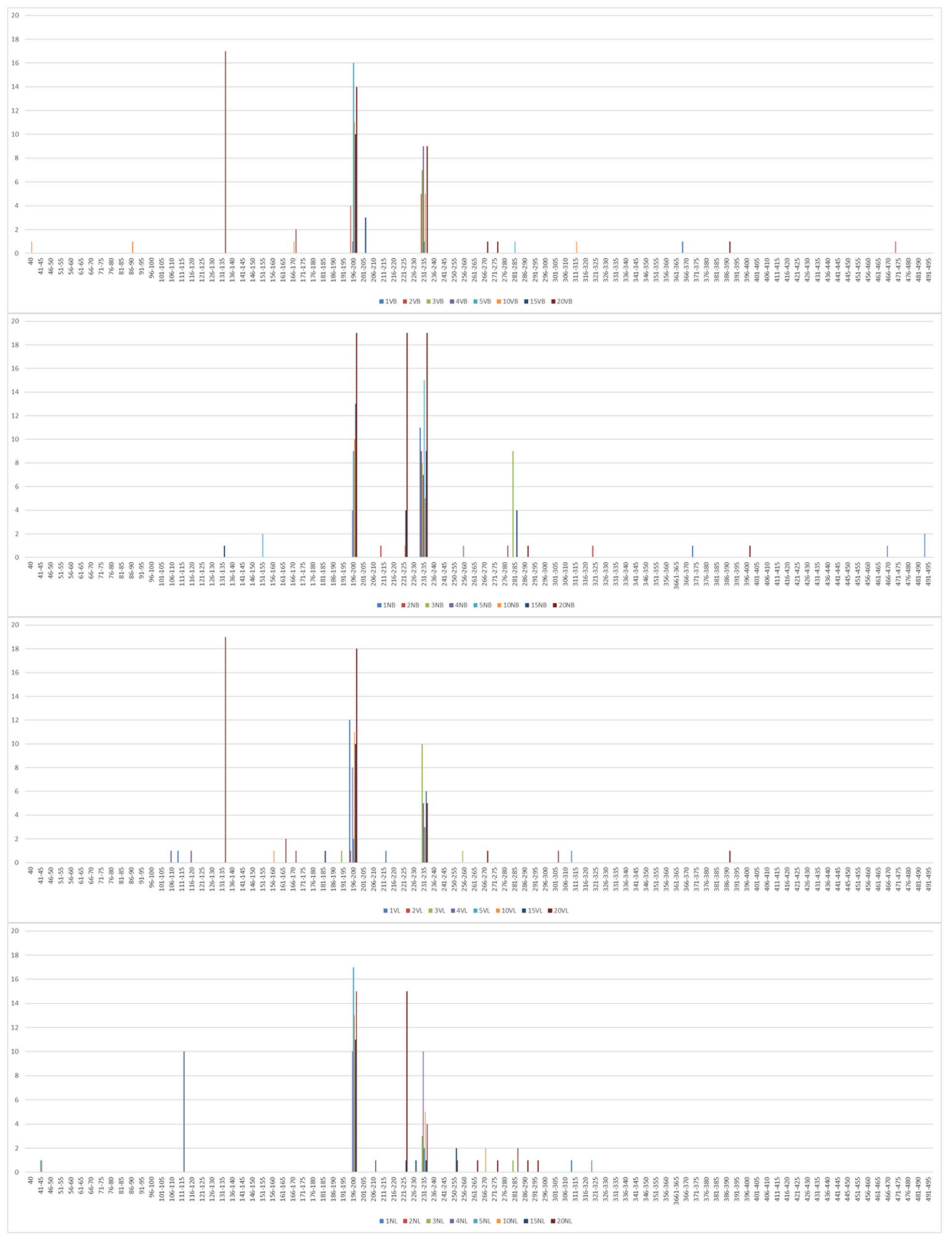
Histogram of influenza mutations by HA amino acid residue. Bars show the number of mutations at each amino acid observed in variants isolated, by sets of five residues as numbered

A spot with a higher mutation rate is called a mutation hotspot. To analyze the differences in the effects of the situations with and without selection pressure of vaccine antibodies on HA of F/98, we defined a special mutation hotspot of the HA after passaging for generations with and without selection pressure of vaccine antibodies by 5 amino acids in a group. After analyzing and comparing with F/98, we found that there were 5 mutation hotspots on HA of the viruses isolated from the trachea with selection pressure (Fig. 3), namely, amino acids 131–135, 166–170, 196–200, 201–205 and 231–235. Among them, the 131–135 mutation hotspot contained K131R in 17 strains; the 166–170 mutation hotspot contained A168T in 3 strains; the 196–200 mutation hotspot contained A198V in 56 strains; the 201–205 mutation hotspot contained N201D in 3 strains; and the 231–235 mutation hotspot contained Q234L in 36 strains. We found that there were 4 mutation hotspots in HA of the viruses isolated from the trachea without selection pressure, namely, amino acids 196–200, 221–225, 231–235 and 281–285. Among them, the 196–200 mutation hotspot contained A198V in 55 strains; the 221–225 mutation hotspot contained M224K in 24 strains; the 231–235 mutation hotspot contained Q234L in 83 strains; the 281–285 mutation hotspot contained L281F in 9 strains; and the 281–285 mutation hotspot contained N285D in 4 strains. The 131–135, 166–170 and 201–205 mutation hotspots were specific to the tracheas with selection pressure; the 221–225 and 281–285 mutation hotspots were specific to the tracheas without selection pressure; and the 196–200 and 231–235 common mutation hotspots were common. The results showed that in the trachea, the selection pressure of vaccine antibodies can generate mutations in the F/98 strain.

In the lung, we found that there were 3 mutation hotspots in HA of the viruses isolated with selection pressure of vaccine antibodies, namely, amino acids 131–135, 196–200 and 231–235. The 131–135 mutation hotspot contained K131R in 18 strains; the 196–200 mutation hotspot contained A198V in 62 strains; and the 231–235 mutation hotspot contained Q234L in 32 strains. There were 4 mutation hotspots in HA of the viruses isolated without selection pressure of vaccine antibodies, namely, amino acids 111–115, 196–200, 221–225 and 231–235. Among them, the 111–115 mutation hotspot contained E114K in 10 strains; the 196–200 mutation hotspot contained A198V in 66 strains; the 221–225 mutation hotspot contained M224K in 16 strains; and the 221–235 mutation hotspot contained Q234L in 25 strains. The number of HA mutation hotspots of the viruses isolated from the lungs with selection pressure of vaccine antibodies was 1 less than that of the viruses isolated from lungs without selection pressure, but the 131–135 mutation hotspot was specific to HA of the viruses from the lungs with selection pressure of vaccine antibodies. The 111–115 and 221–225 mutation hotspots were specific to HA of the viruses from the lungs without selection pressure of vaccine antibodies, while all the HA genes of the viruses isolated from the lungs with and without selection pressure of vaccine antibodies had the 196––200 and 231–235 mutation hotspots. In summary, F/98 generated 3 specific hotspots affected by the selection pressure of vaccine antibodies, namely, amino acids 131–135, 166–170 and 201–205; F/98 also generated 3 specific hotspots without being affected by the selection pressure of vaccine antibodies, namely, amino acids 111–115, 221–225 and 281–285; and F/98 generated the 196–200 and 231–2352 hotspots with and without the selection pressure of vaccine antibodies. These results showed that the HA protein of H9N2 has its own specific evolution and mutation strategies when escaping from the selection pressure of vaccine antibodies of the host.

### The mutation position and distribution features in the natural environment of the HA gene of H9N2 subtype avian influenza virus with selection pressure of vaccine antibodies

**Fig. 4:**
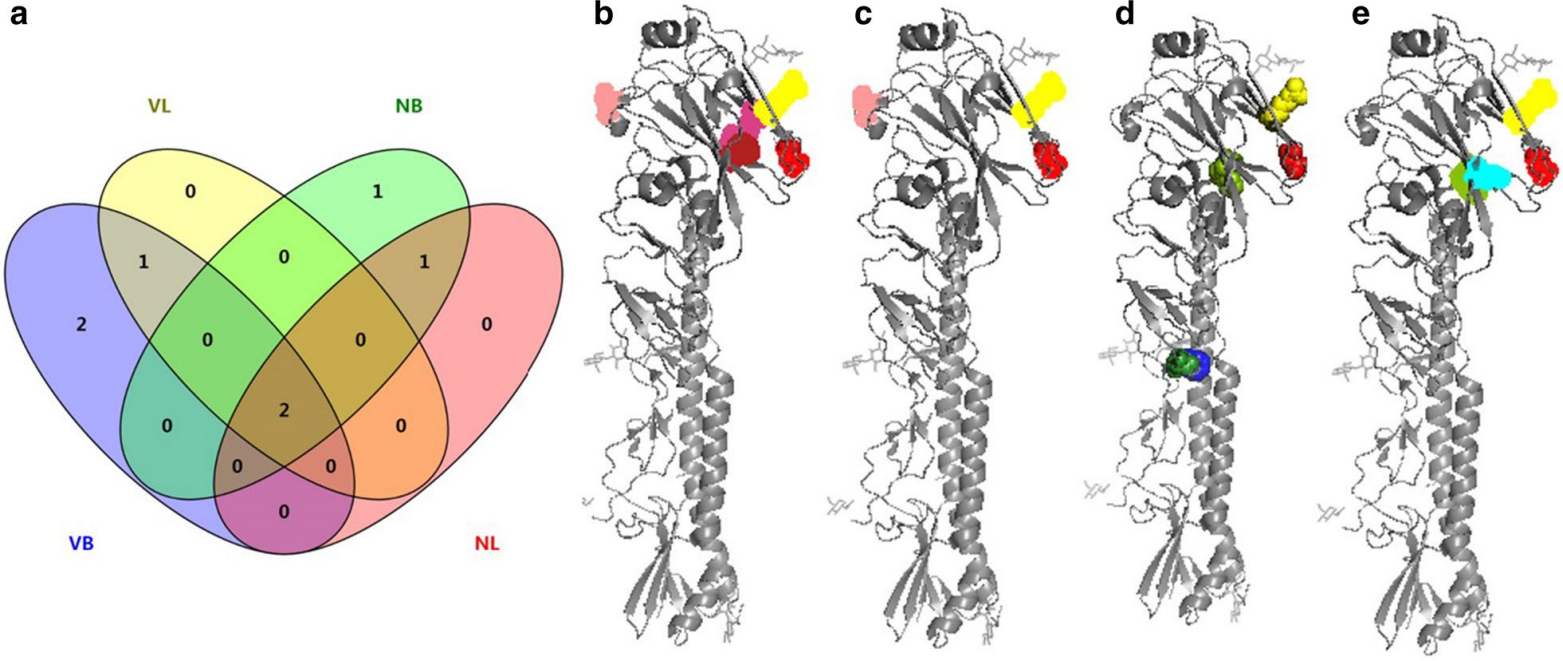
Most frequently occurring mutations. **a** The statistics of the most frequently occurring variants. **b**–**e** Location of the most frequently occurring variants on the 3D structure. **b** VB; **c** VL; **d** NB; and **e** NL. Residues: 198: red; 234: yellow; 131: salmon; 168: firebrick; 201: warm pink; 114: cyan, 224: split pea; 281: forest; and 285: blue

The previous results show that the HA gene of the F/98 strain virus with selection pressure of vaccine antibodies generated specific mutation hotspots in some areas. To understand the features of these specific mutations, we marked the areas for HA amino acid sequence mutation hotspots of quasispecies viruses serially passaged from generation to generation with and without selection pressure of vaccine antibodies on the 3D structural model of the HA protein and then analyzed the ratio of them observed in natural isolated strains. We found that the HA gene mutations K131R, A168T, A198T and N201D of quasispecies viruses serially passaged from generation to generation with selection pressure of vaccine antibodies were all located in the head of HA protein; the mutations HA gene E114K, A198T, M224K and L234Q of quasispecies viruses serially passaged from generation to generation without selection pressure of vaccine antibodies were located in the head of HA protein, while L281F and N285D were located in the neck of the HA protein (Fig. 4).

**Fig. 5:**
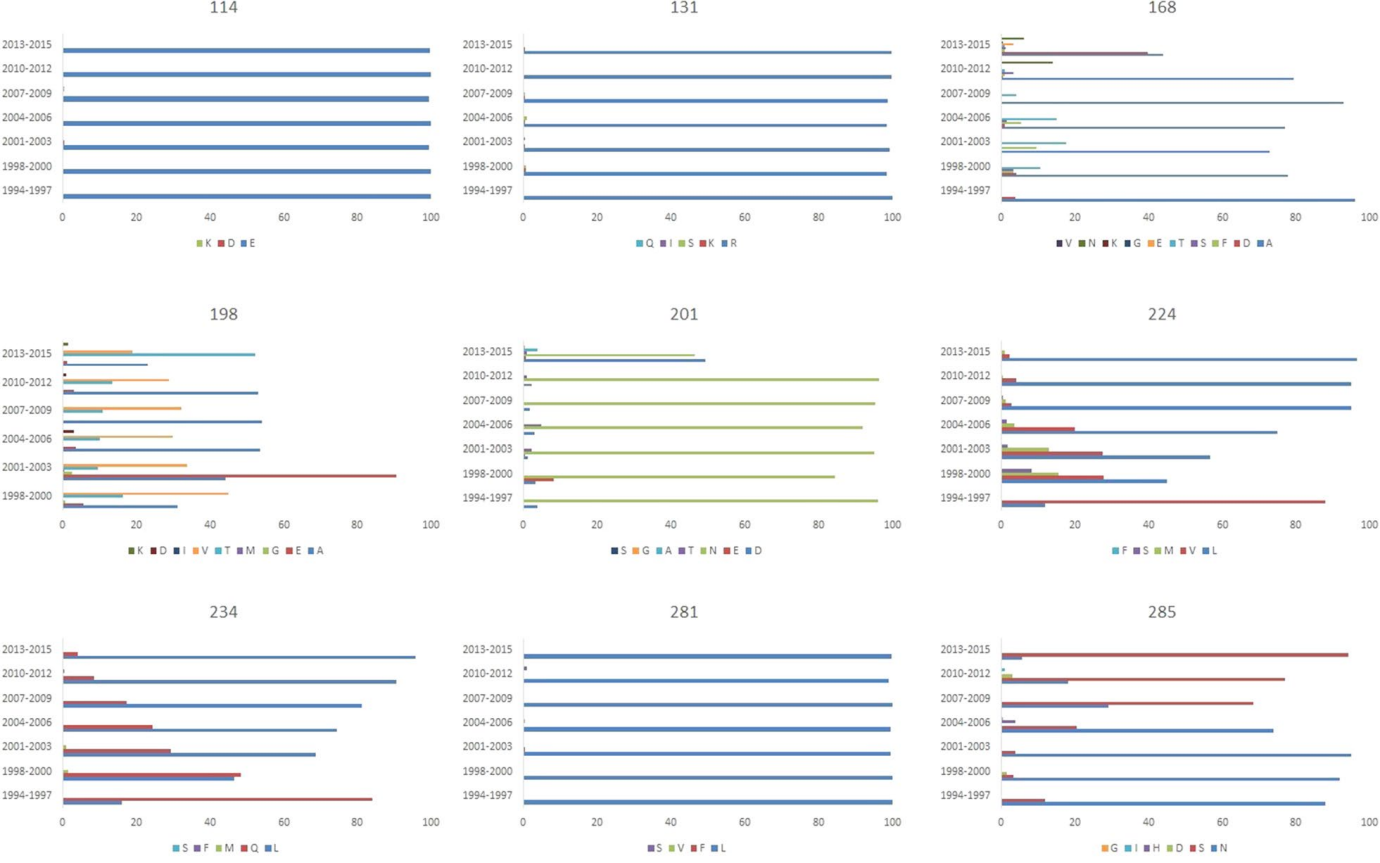
Hotspots on HA of wild-type H9N2 strains. The HA genes were loaded from GenBank. The Y axis represents the years when the strains were isolated

To analyze the distribution of these mutation hotspots in naturally isolated strains, we analyzed the residues 114, 131, 168, 198, 201, 224, 234, 281 and 285 of the HA genes of the H9 subtype avian influenza virus released in GenBank from 1994 to 2015. It was found that the major amino acid at the positions 114, 131 and 281 on the HA genes of natural isolated strains was E, R, and L, respectively (accounting for > 99.5%), while the positions 168, 198, 201, 224, 234, and 285 had multiple amino acids existing in the HA gene of the H9 subtype avian influenza viruses isolated over many years. These mutations also existed in the 131–135 and 166–170 (166–170) hotspots, which were specific to the HA gene from viruses serially passaged from generation to generation with selection pressure of vaccine antibodies. The HA genes of viruses that were serial passaged from generation to generation with and without selection pressure of vaccine antibodies all had the 196–200 and 231–235 (198, 224, 234) mutation hotspots, and the mutation hotspot of the HA genes of viruses that were serially passaged from generation to generation with selection pressure of vaccine antibodies was amino acids 281–285 (281 and 285) (Fig. 5). Accordingly, mutation of the positions 131, 168 and 201 of natural isolated strains was related to the immunization status of the host, while mutation of the positions 224, 281 and 285 was related to the continuous replication and dissemination of the avian influenza virus in the host, and mutation of the positions 198 and 234 may be related to both factors.

### The specific amino acid variation of the H9N2 subtype avian influenza virus F/98 strain with selection pressure of vaccine antibodies in chickens

After serial passaging from generation to generation for H9N2 subtype avian influenza virus F/98, in addition to mutation of the HA gene, other genes had also undergone corresponding changes. After serial passaging, the quasispecies viruses from the tracheas and lungs of the twentieth-generation chickens were isolated from plaques. Then, the NA, PB2, PB1, PA, NP, M and NS genes of the progeny viruses were sequenced.

### Ten amino acids lost in NA of the F/98 strain of the H9N2 subtype avian influenza virus with selection pressure of vaccine antibodies

NA is endowed with sialidase activity, which cleaves host cell sialic acid and generates new virus particles. NA is related to virus germination and release (Knipe and Howley 2013). Compared with those in F/98, in the 44 strains of the twentieth generation isolated from trachea and lung with selection pressure of vaccine antibodies, ten amino acids (67–76) of NA were lost, while the virus without selection pressure had not undergone mutation and deletion (Table 5). The selection pressure of vaccine antibodies led to the NA gene losing amino acids 67–76, and their functions were further studied.

**Table 5:** List of mutations of the genes other than HA in progeny strains

Gen	Tissue	Immunized	Nonimmunized
NA	Trachea	Amino acids 67–76 deletion (20/20)	–
	Lung	Amino acids 67–76 deletion (24/24)	–
PB2	Trachea	D256N (1/20), R318K (13/20), R355K (20/20)	R318K (19/19)
	Lung	R318K (19/24), R355K (24/24), K526R (1/24), A622V (2/24)	I63M (1/15), R318K (15/15)
PB1	Trachea	I397V (1/20)	I517V (1/19)
	Lung	–	–
PA	Trachea	–	R185K (1/19), S190F (1/19), E533G (1/19), F646S (1/19), C693G (1/19)
	Lung	F46L (1/24), V127I (1/24), L132I (1/24), E319V (1/24)	V554I (1/15)
NP	Trachea	–	V186I (19/19), L466I (9/19)
	Lung	–	V186I (15/15), L466I (14/15)
NS	Trachea	–	L33Q (1/19), L77I (19/19)
	Lung	P85L (1/24)	L77I (15/15), V84M (1/15)
M1	Trachea	L28P (1/20)	F62L (2/19), R256H (1/19)
	Lung	–	–
M2	Trachea	–	–
	Lung	R304G (2/24)	A209T (1/15)

### Analysis of polymerase gene mutation of the twentieth-generation quasispecies viruses serially passaged from generation to generation with and without selection pressure of vaccine antibodies

Since the polymerase of avian influenza virus lacks proofreading function, it leads to the avian influenza virus constantly mutating, which occurs on not only the surface genes of avian influenza virus but also the internal genes. To study the effect of the selection pressure of vaccine antibodies on F/98 internal genes, the internal genes of the twentieth-generation quasispecies viruses (78 strains) were sequenced.

**Fig. 6:**
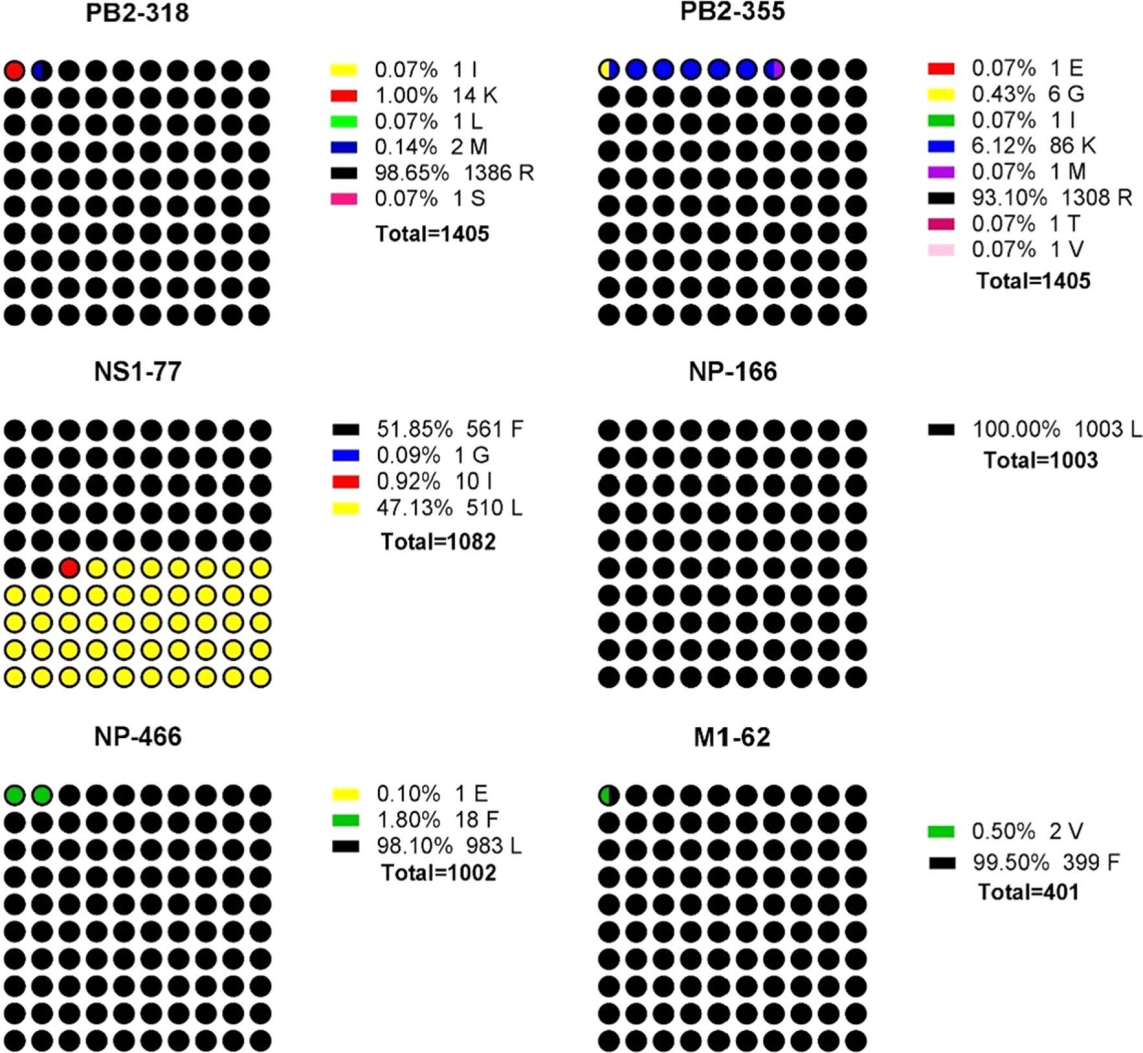
Mutation rate of wild-type strains in NCBI. The genes were loaded from GenBank

In the 20 strains isolated from the tracheas of chickens with selection pressure, PB2 of 1 strain (5%) had a D256N mutation, PB2 of 13 strains (65%) had an R318K mutation and PB2 of 20 strain (100%) had an R355K mutation; in the 24 strains isolated from the tracheas of chickens with selection pressure, PB2 of 19 strains (79.1%) had an R318K mutation, PB2 of 24 strains (100%) had an R355K mutation PB2 of 1 strain (5.2%) had a K526R mutation, and PB2 of 2 strains (10.5%) had an A622V mutation. In the 19 strains of viruses isolated from the tracheas of chickens without selection pressure, all the PB2 proteins of the 19 strains (100%) had an R318K mutation; in the 15 strains isolated from the tracheas of chickens without selection pressure, PB2 of 1 strain (6.6%) had an I63M mutation and PB2 of the 15 strains (100%) had an R318K mutation (Table 5). After analyzing the gene data of wild-type strains in the NCBI avian influenza virus database, the position 318 of PB2 in the viruses isolated from the natural environment is an R in the majority and a K in 1%, while the position 355 of PB2 in the viruses isolated from the natural environment is an R in the majority and a K in 6.25% (Fig. 6). The results showed that all the PB2 genes of the viruses isolated from tracheas and lungs of the twentieth-generation chickens with selection pressure had R355K mutations, and 72% of the PB2 genes from isolated strains had R318K mutations. The PB2 gene of 100% of isolated strains from the tracheas and lungs of the twentieth-generation chickens without selection pressure had R318K mutations, but there was no R355K mutation, which signified that the R355K mutation in the PB2 genes was the result of the selection pressure of vaccine antibodies on F/98. The R318K mutation in the PB2 gene appeared with and without selection pressure of vaccine antibodies, although the PB2 gene mutation rate of the isolated strains after serial passaging was lower than that without selection pressure of vaccine antibodies.

Compared with the amino acid sequence of PB1 of F/98, there were no mutations detected for the viruses isolated from the lungs with and without selection pressure of vaccine antibodies. In PB1 of 20 strains of quasispecies viruses isolated from the trachea in the twentieth generation after serial passaging with selection pressure of vaccine antibodies, there was only an I397V mutation in PB1 of 1 strain; in PB1 of 19 strains of quasispecies viruses isolated from the tracheas in the twentieth generation after serial passaging without selection pressure, there was only an I517V mutation in PB1 of 1 strain virus (Table 5). The strains with I397V and I517V mutations in the PB1 genes were not advantageous, and their biological significance was not manifested in this study.

In terms of PA genes, compared with the amino acid sequence of the PA gene of the F/98 strain virus, there were no mutations detected for the 20 strains isolated from the trachea in the twentieth generation after serial passaging with selection pressure. For the 24 strains isolated from the lung in the twentieth generation after serial passaging with selection pressure, 1 strain had an F46L mutation, 1 strain had a V127I mutation, 1 strain had an L132I mutation and 1 strain had an E319V mutation. For the 19 strains isolated from the trachea in the twentieth generation after serial passaging without selection pressure, 1 strain had an R185K mutation, 1 strain had an S190F mutation, 1 strain had an F646S mutation and 1 strain had a C693G mutation. For the 15 strains isolated from the lungs in the twentieth generation after serial passaging without selection pressure, 1 strain had a V554I mutation (Table 5). These mutations detected from the PB1 and PA proteins independently existed in one strain of virus. Since avian influenza virus undergoes error-prone replication, we were not sure whether these mutations were related to immunization, so their significance remains to be further studied.

### Analysis of NP protein, M protein and NS protein mutations of the twentieth generation quasispecies viruses after serial passaging with and without selection pressure

The NP protein of avian influenza virus is able to combine with the RNA of the virus genome and form a ribonucleoprotein (RNP) complex with RNA polymerase PB2, PB1, and PA to assist the virus in realizing transcription and replication (Baudin et al. 1994). The smallest gene section of influenza A virus is NS, which codes the two proteins NS1 and NS2 (Dundon and Capua 2009; Lin et al. 2007; Selman et al. 2012; Vasin et al. 2014). The NS1 protein is related to the toxicity of influenza A virus, which is engaged in replication of influenza virus and regulation of viral protein synthesis and affects the morphological structure of virus particles, as well as prohibits the host immune response and apoptosis of cells (Khaperskyy and McCormick 2015).

For NP, compared with the F/98 sequence, there were no mutations detected for viruses isolated from the tracheas (20 strains) and lungs (24 strains) in the twentieth generation after serial passaging with selection pressure. For the 19 strains isolated from the trachea in the twentieth generation after serial passaging without selection pressure, all 19 strains had a V186I mutation, and 9 strains had an L466I mutation. For the 15 strains isolated from the trachea in the twentieth generation after serial passaging without selection pressure, all 15 strains had a V186I mutation, and 14 strains had an L466I mutation, which shows that the V186I and L466I mutations were related to the mutation-prone nature of the virus. Similarly, this result also indicates that the selection pressure of vaccine antibodies limits the NP gene to generate the V186I and L466I mutations (Table 5). For the M gene, compared with the F/98 sequence, for the 20 strains isolated from the trachea in the twentieth generation after serial passaging with selection pressure, the M1 gene of 1 strain had an L28P mutation, and the M1 gene of 2 strains of the 24 strains isolated from the lung had an R304G mutation. For the 19 strains isolated from the trachea in the twentieth generation after serial passaging without selection pressure, the M1 gene of 2 strains had an F62L mutation, and the M2 gene of 1 strain had an R256H mutation; for the 15 strains isolated from the lung, the M2 gene of 1 strain had an A209T mutation (Table 5). For the NS gene, compared with the F/98 sequence, there were no mutations detected in the strains isolated from the trachea; for the 24 strains isolated from the lung, the NS gene of 1 strain had a P85L mutation. For the 19 strains isolated from the trachea in the twentieth generation after serial passaging without selection pressure, the NS gene of 1 strain had an L33Q mutation, and the NS1 gene of 19 strains had an L77I mutation; for the 15 strains isolated from the lung, the NS gene of 15 strains had an L77I mutation, and the NS gene of 1 strain had a V84M mutation (Table 5). After analyzing the gene data for wild-type strains in the NCBI influenza database, it was found that the V186I and L466I mutations of NP genes do not exist in wild type, while the L77I mutation in the NS gene accounted for only 0.92% (Fig. 6). The V186I and L466I mutations of the NP gene and the L77I mutation of the NS gene occur in only the viruses passaging from generation to generation without the selection pressure of vaccine antibodies, which indicates that the selection pressure of vaccine antibodies was less effective for NS and NP than for other viral proteins.

### Selection pressure of vaccine antibodies regulated the infection of the F/98 strain virus

**Fig. 7:**
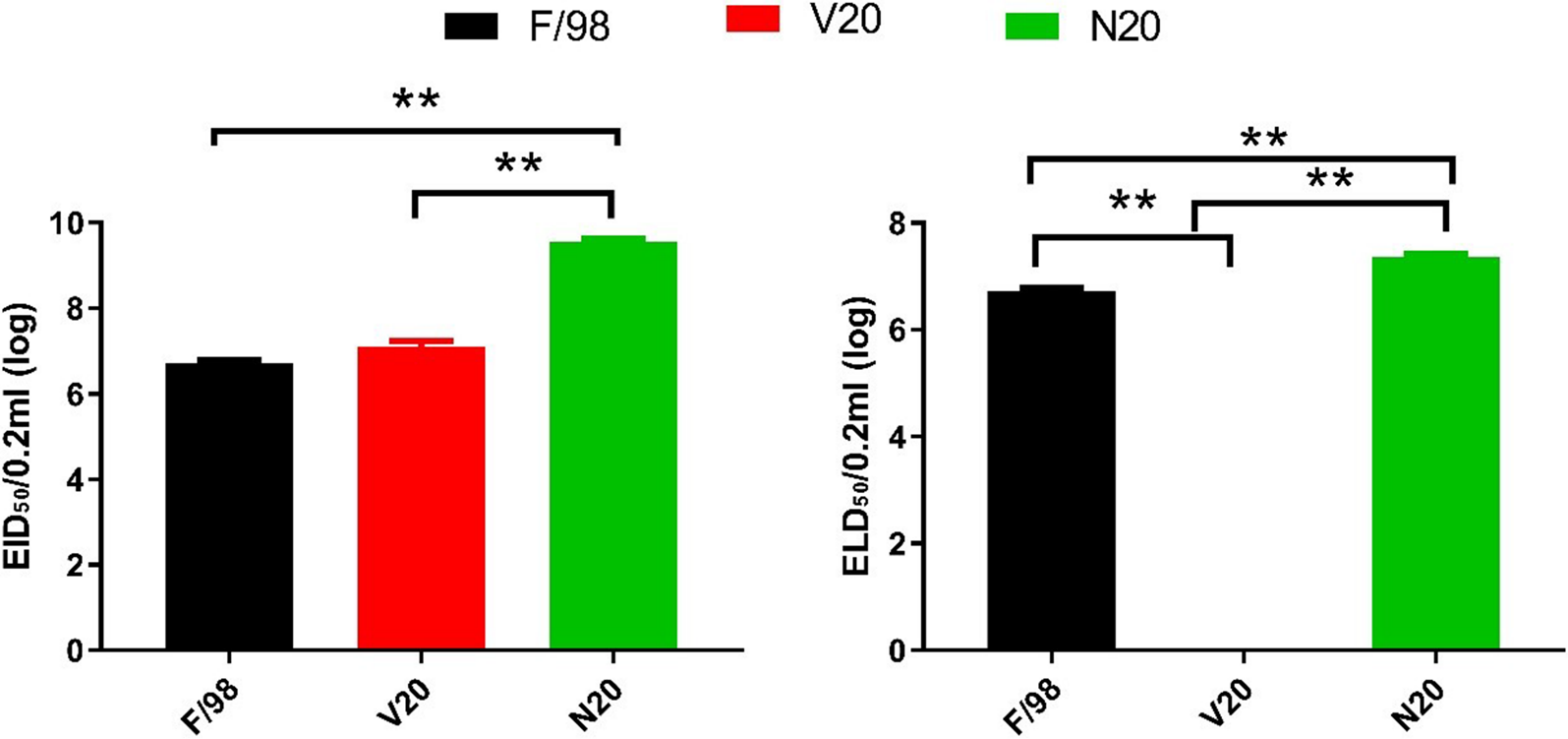
EID_50_ and ELD_50_of the progeny strains. **, significant difference, P < 0.01

To explore the effect of the selection pressure of vaccine antibodies on the infection of F/98, the study selected progeny viruses to detect the EID_50_ and ELD_50_, according to the mutations of each gene. For the viruses with and without selection pressure of vaccine antibodies, we selected 5 strains each, and the mutations in each strain were as follows (seen in Table 6): V1: HA (K131R, A198V, Q234L), NA (67–76 amino acid deletion), PB2 (R318K, R355K); V2: HA (K131R, A168T, A198T, L234Q), NA (67–76 amino acid deletion), PB2 (R318K); V3: HA (K131R, N201D, A198T, L234Q), NA (67–76 amino acid deletion); V4: HA (K131R, A198T, L234Q), NA (67–76 amino acid deletion), PB2 (R318K, R355K); V5: HA (K131R, A198T, L234Q), NA (67–76 amino acid deletion); N1: HA (A198V, M224K, Q234L, L281F, N285D), PB2 (R318K), NP (V186I, L466I), NS1 (L77I), M1 (F62L); N2: HA (A198V, M224K, Q234L, N285D), PB2 (R318K), NP (V186I, L466I), NS1 (L77I), M1 (F62L); N3: HA (A198V, M224K, Q234L, L281F, N285D), PB2 (R318K), NP (V186I, L466I), NS1 (L77I), M1 (F62L); N4: HA (E114K, A198V, M224K, Q234L, L281F, N285D), PB2 (R318K), NP (V186I, L466I), NS1 (L77I), M1 (F62L); and N5: HA (A198V, M224K, Q234L, L281F, N285D), PB2 (R318K), NP (V186I, L466I), NS1 (L77I), M1 (F62L). The EID_50_ values of progeny viruses V1–V5 with selection pressure of vaccine antibodies were within 6.9–7.2; in the testing process of ELD_50_, within 120 h after infection, there were no cases of infectious embryo death; the EID_50_ values of the progeny viruses N1–N5 without selection pressure of vaccine antibodies were within 7.2–7.5 (shown in Fig. 7). Compared with that of F/98, there was no obvious change in the EID_50_ of the viruses with selection pressure of vaccine antibodies (increased by 1.9 times), while the EID_50_ of the viruses without selection pressure of vaccine antibodies was 794 times more than that of F/98 and 397 times more than that of the viruses with selection pressure of vaccine antibodies, so their infection ability rose obviously (P < 0.01). Compared with that of F/98, the ELD_50_ of V20 with selection pressure of vaccine antibodies was 0, and it was not fatal; its reduced ELD_50_ was obvious (P < 0.01), while the EID_50_ of N20 was 6.3 times greater than that of F/98, and the ELD_50_ was obviously increased (P < 0.01). These results show that the selection pressure of vaccine antibodies can regulate the infectious ability of H9N2 and the fatality of the chick embryo.

**Table 6:** ELD_50_ and ELD_50_ of the progeny strains

Immune status	Tissue	Strains	Mutations						EID_50_	ELD_50_
			HA	NA	PB2	NP	NS1	M1		
Immunized	Trachea	V1	K131R, A198T, L234Q	Amino acids 67–76 deletion	R318K, R355K	–	–	–	6.9	0
Immunized	Lung	V2	K131R, A168T, A198T, L234Q	Amino acids 67–76 deletion	R318K	–	–	–	7	0
Immunized	Trachea	V3	K131R, N201D, A198T, L234Q	Amino acids 67–76 deletion	–	–	–	–	7.2	0
Immunized	Lung	V4	K131R, A198T, L234Q	Amino acids 67–76 deletion	R318K, R355K	–	–	–	7	0
Immunized	Lung	V5	K131R, A198T, L234Q	Amino acids 67–76 deletion	–	–	–	–	7	0
Nonimmunized	Trachea	N1	A198V, M224K, Q234L, L281F, N285D	–	R318K	V186I, L466I	L77I	F62L	9.5	7.4
Nonimmunized	Trachea	N2	A198V, M224K, Q234L, N285D	–	R318K	V186I, L466I	L77I	F62L	9.6	7.3
Nonimmunized	Lung	N3	A198V, M224K, Q234L, L281F, N285D	–	R318K	V186I, L466I	L77I	F62L	9.1	7.2
Nonimmunized	Lung	N4	E114K, A198V, M224K, Q234L, L281F, N285D	–	R318K	V186I, L466I	L77I	F62L	9.8	7.5
Nonimmunized	Trachea	N5	A198V, M224K, Q234L, L281F, N285D	–	R318K	V186I, L466I	L77I	F62L	9.4	7.4

## Discussion

H9 subtype avian influenza virus was divided into 4 main evolutionary branches: h9.1–h9.4 (Jiang et al. 2012). In the 1990s, H9N2 subtype avian influenza virus was isolated and found in mainland China for the first time; by now, more than 20 years have passed. During the process, the H9N2 subtype avian influenza virus has been in constant mutation, and there are many branches (Kang et al. 2006; Liu et al. 2003; Ma et al. 2018; Mancera Gracia et al. 2018; Matrosovich et al. 2001; Mok et al. 2011; Ninomiya et al. 2002; Wu et al. 2008; Xu et al. 2008; Zhang et al. 2009, 2012). In 2007 and before, the H9N2 subtype avian influenza virus in China belonged to the evolved branches of h9.4.2.1–h9.4.2.4; among them, F/98 was in line with the F/98-like branch in the system (Sun and Liu 2015; Sun et al. 2010). In 2007–2010, h9.4.2.5 had become the advantageous popular strain (Jiang et al. 2012; Zhang et al. 2015); after 2010, the h9.4.2.6 virus represented by A/chicken/Guangdong/FZH/2011 also appeared in southern China (Gu et al. 2017). To control the infection and transmission of the H9N2 subtype avian influenza virus, a vaccination measure was taken in mainland China to control and prevent the virus (Jin et al. 2018). At present, the H9N2 subtype avian influenza virus is still pervasive in most provinces of China. According to relevant reports, there are still infectious cases despite preimmunization measures producing high levels of antibodies (Zhang et al. 2015). This finding shows that the H9N2 subtype avian influenza virus can still duplicate within the body of poultry with antibodies, and there are mutations for the virus.

Previous studies have shown that serial passaging of the influenza virus in nonimmunized animals can lead to increased pathogenicity and transmission of the virus (Guarnaccia et al. 2013; Li et al. 2014; Murcia et al. 2012; Pavulraj et al. 2015). Moreover, the infectivity of virus serially passaged without selection pressure of vaccine antibodies also increases (Jin et al. 2018; Shanmuganatham et al. 2016). In this experiment, the infectivity of progeny virus of the F/98 strain after serial passaging for more than 20 generations was obviously increased (P < 0.01), which was in line with the previous research results. However, the EID_50_ of the progeny virus of the F/98 strain after serial passaging for more than 20 generations in SPF chickens was not obviously increased, and the progeny virus was not able to kill chick embryos. We hypothesize that the selection pressure of vaccine antibodies forces the F/98 strain to evolve in the direction of symbiosis with the host chicken.

Avian influenza virus has strong evolutionary ability because of its high rate of gene mutation (Knipe and Howley 2013). In the host body, the virus generates some adaptive mutations. To escape from the selection pressure of vaccine antibodies of the host, F/98 generated a series of mutations that helped to adapt to the selection pressure of vaccine antibodies. Compared to that of F/98, the average HI titer of the second generation progeny viruses isolated from trachea and lung tissues with selection pressure of vaccine antibodies was decreased by 4.7 and 5.3 times, respectively, and more than 60% of the progeny viruses had generated antigen mutations. As a comparison, among the virus serially passaged without selection pressure of vaccine antibodies, antigenic variation was observed for less than 50% of the quasispecies strains in the fifth generation of progeny viruses isolated from the trachea or lung tissues. Therefore, we conclude that the selection pressure of vaccine antibodies accelerated the antigen mutation process of H9N2 subtype avian influenza virus.

In the virus–host interaction process, the dynamic process of host-influenced adaptation occurs (Knipe and Howley 2013). After testing the sequences of each quasispecies virus, we found that some mutations appeared in quasispecies viruses after one passage but they were not detected after additional passages. In viruses passaged 5 times, although the A198V mutation could be stably passed down to subsequent generations, such cases still appeared; for example, in 1VL, 2VL, 4VL and 5VL, the A198V mutation was obvious, but amino acid 198 in 3VL virus was not mutated. Moreover, there were 2 amino acid mutations in the HA protein for both the 1NL and 3NL viruses, but there was no mutation detected for the HA protein in the 2NL virus.

HA protein is the surface protein for avian influenza virus with an antigen epitope used to induce neutralizing antibody production in the host (Lee et al. 2005). Regarding mutation of the antigen, we discovered after sequencing that there were K131R, A168T, A198V, N201D and Q234L mutation hotspots in the HA genes of progeny viruses with selection pressure of vaccine antibodies and there were E114K, M224K, A198V, Q234L, L281F and N285D mutation hotspots in the HA genes of progeny viruses without selection pressure of vaccine antibodies. This result indicates that the selection pressure of vaccine antibodies makes the F/98 strain generate specific HA gene mutations, namely, K131, A168T and N201D. After annotating the H9 molecular crystal, we determined that K131R, A168T, A198V, N201D, and Q234L are located near the head area of HA (Fig. 4). Mutation of the 131 and 198 hotspots of the HA gene of H9N2 subtype avian influenza virus affects the binding of antibody to the viral surface antigen, while the mutation 201D can reduce the affinity between the virus and the avian receptor (Ilyushina et al. 2004; Kaverin et al. 2004; Lee and Suarez 2005; Zhang et al. 2015). It should be mentioned that after serial passaging in the host chicken, the Q234L mutation of the HA gene (H3 No. 226) can be generated regardless of the selection pressure of vaccine antibodies, while the Q234L mutation makes the virus able to affect mammals (Chen et al. 2013; Zhang et al. 2015). This result was of great significance for public health and should be highlighted by the public.

The NA protein is related to virus seeding from cells, while the infectivity of the virus is related to the balanced relationship between HA and NA (Durrant et al. 2016; Knipe and Howley 2013). The amino acid losses of the HA protein in H5N2 and H2N2 subtype avian influenza viruses can enhance the infectivity of the virus (Munier et al. 2010; Sorrell et al. 2010). There were 10 amino acid losses in the NA protein ranging from positions 67–76 for H9N2 avian influenza virus with selection pressure of vaccine antibodies, while the infectivity of viruses with selection pressure of vaccine antibodies after serial passaging was obviously enhanced. Whether this result was related to the loss of the 10 amino acids remains to be further studied.

PB2 is part of the polymerase trimer complex subunit of influenza virus. There were two mutation hotspots, R318K and R355K, in the PB2 gene of quasispecies viruses obtained with selection pressure of vaccine antibodies. On PB2 of the viruses obtained without selection pressure of vaccine antibodies, there was only one amino acid mutation (R318K), and it existed on most quasispecies viruses. Mutation on the RNP complex will increase the infectivity and host range of influenza viruses, such as 588V, E627K, G685R and D701N on PB2 (Song et al. 2011; Wei and Liu 2017; Xiao et al. 2016; Zhang et al. 2015). PB2 can generate the specific and stable mutations R355K with R355K. Infectivity and host range changes for influenza viruses and other biological significance remain to be further studied.

PA and PB1 are important components of the RNA polymerase complex of influenza virus (Kaverin et al. 2004). After sequence analysis, it was found that there were 4 mutations for progeny viruses isolated from lungs with selection pressure of vaccine antibodies. There was only 1 mutation for progeny viruses isolated from lungs without selection pressure of vaccine antibodies. In contrast, no PA mutation was detected for progeny viruses isolated from tracheas with selection pressure of vaccine antibodies. Six mutations were detected for progeny viruses isolated from tracheas without selection pressure of vaccine antibodies. NS encoded by influenza virus can restrict the secretion of host interferon (Hale et al. 2008; Marc 2012). There were 2 mutations for progeny viruses isolated after serial passaging with selection pressure of vaccine antibodies. There were 4 mutations on NS for progeny viruses isolated after serial passaging without selection pressure of vaccine antibodies, and L77I existed on all the progeny viruses. NP, RNA polymerase complex and RNA sections of viruses are the core of influenza virus particles (Stadt et al. 2005). There was no mutation on NP with selection pressure of vaccine antibodies, while there were two high-frequency mutations on NP without selection pressure of vaccine antibodies, namely, V186I (19/19) and L466I (9/19). Therefore, we think that H9N2 generates surface antigen mutations with the selection pressure of vaccine antibodies to adapt to the host; moreover, its internal genes also generate corresponding mutations. The selection pressure of vaccine antibodies regulated the internal genes of H9N2 subtype avian influenza virus.

## Data Availability

The strains used in this study are stored at the Key Open Lab of Animal Infectious Diseases of the Agricultural Ministry (Yangzhou, China). The genomes data of F/98 could be obtained from Genbank (Accession number: PB2, AY253750; PB1, AY253751; PA, AY253752; HA, AF743216; NP, AY253753; NA, AY253754; M, AY253755; NS, AY253756).

## Abbreviations

SPF: Specific pathogen-free
MDCK: Madin–Darby canine kidney
DMEM: Dulbecco’s modified Eagle medium
HI: Hemagglutination inhibition
PBS: Phosphate-buffered saline
HAU: Hemagglutinating units
RBCs: Red blood cells
RDE: Receptor-destroying enzyme
EID_50_: 50% egg infectious dose
ELD_50_: 50% embryo lethal dose
